# Experimental VILI begins with subpleural lung lesions

**DOI:** 10.1186/cc13473

**Published:** 2014-03-17

**Authors:** C Chiurazzi, M Gotti, M Amini, C Rovati, M Brioni, G Rossignoli, A Cammaroto, C Bacile, S Luoni, K Nikolla, C Montaruli, T Langer, D Dondossola, M Cressoni, L Gattinoni

**Affiliations:** 1 Fondazione IRCCS Ca Granda, Milan, Italy

## Introduction

We developed an experimental model of VILI, ventilating piglets at a strain (TV/FRC) >2.5. It is possible that lung inhomogeneities act as stress raisers within the lung parenchyma, locally multiplying pressures. In a healthy lung the pleural surface, vessels and bronchi are detected as natural lung inhomogeneities. We studied the development of VILI with CT scan to determine where the first lung lesion developed.

## Methods

Piglets were sedated, orotracheally intubated and instrumented with arterial and central venous catheter and urinary catheter. The whole study was performed in the animal CT scan facility, which was equipped as an iCu, and CT scan was performed every 3 hours or if respiratory parameters (plateau/peak pressure) changed. We defined as new lesion lung regions the appearance of poorly inflated/not inflated lung regions not present in the previous CT scan image. We select the first CT scan in which a relevant number of new lesions appeared (>15) and manually delineated the lesions; the lesions were classified as close/not close to the pleural surface.

## Results

**Figure 1:**
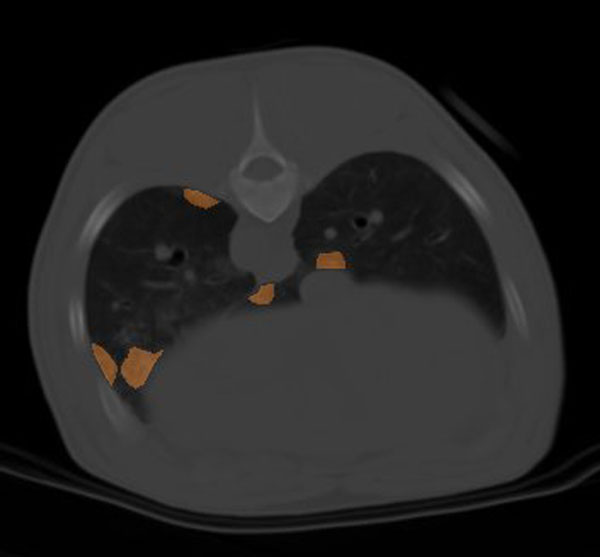


Six piglets were studied (22 ± 8 kg) that were ventilated with a TV of 750 ± 71 ml (41 ± 1 ml/kg) up to development of VILI, defined radiologically as infiltrates present in all pulmonary fields at CT scan plus development of lung edema. In the first CT scan where lesions appeared, a median of 28 lesions (IQ range 22 to 30) were present. Of these lesions, 18 (17 to 22) (72%) were located near the pleura and nine (6 to 11) (28%) near vessels/bronchi. See Figure 1.

## Conclusion

In an experimental model of VILI the first lung lesions appear below the pleural surface. Mutiple nonmutually exclusive possible explanations are possible: the pleural surface acts as a stress raiser; the mechanical friction of the lung with the ribs at very high tidal volume leads to parenchimal injury; and the lung skeleton is a fan-like structure starting from the hilum and going to the pleural surface, leading to increased stress/strain of the subpleural regions.

